# Solution structure and binding specificity of the p63 DNA binding domain

**DOI:** 10.1038/srep26707

**Published:** 2016-05-26

**Authors:** Andreas Enthart, Christian Klein, Alexander Dehner, Murray Coles, Gerd Gemmecker, Horst Kessler, Franz Hagn

**Affiliations:** 1 Center for Integrated Protein Science Munich (CIPSM) at the Department Chemie, Technische Universität München, 85747 Garching, Germany; 2 Institute for Advanced Study, Technische Universität München, 85748 Garching, Germany; 3 Helmholtz Zentrum München, Institute of Structural Biology, 85764 Neuherberg, Germany; 4 Roche Pharmaceutical Research & Early Development, Roche Innovation Center Zurich, CH-8952 Schlieren, Switzerland; 5 Department of Protein Evolution, Max-Planck-Institute for Developmental Biology, Tübingen, Germany

## Abstract

p63 is a close homologue of p53 and, together with p73, is grouped into the p53 family of transcription factors. p63 is known to be involved in the induction of controlled apoptosis important for differentiation processes, germ line integrity and development. Despite its high homology to p53, especially within the DNA binding domain (DBD), p63-DBD does not show cooperative DNA binding properties and is significantly more stable against thermal and chemical denaturation. Here, we determined the solution structure of p63-DBD and show that it is markedly less dynamic than p53-DBD. In addition, we also investigate the effect of a double salt bridge present in p53-DBD, but not in p63-DBD on the cooperative binding behavior and specificity to various DNA sites. Restoration of the salt bridges in p63-DBD by mutagenesis leads to enhanced binding affinity to p53-specific, but not p63-specific response elements. Furthermore, we show that p63-DBD is capable of binding to anti-apoptotic BclxL via its DNA binding interface, a feature that has only been shown for p53 so far. These data suggest that all p53 family members - despite alterations in the specificity and binding affinity - are capable of activating pro-apoptotic pathways in a tissue specific manner.

Due to its prominent role in apoptosis, the tumor suppressor protein p53 has been called the ‘guardian of the genome’^1^ and studied in great detail for several decades^2^,^3^. However, the discovery of protein homologues, named p63^4^ and p73^5^, led to the concept of a family of transcription factors. The high level of sequence similarity in the DNA binding domain (DBD) allows p63 and p73 to trans-activate p53-responsive genes resulting in cell-cycle arrest and apoptosis^6^,^7^. While p53 has specialized on tumor suppression, its “ancestor”^8^,^9^,^10^ p63 plays a predominant role in the regulation of epithelial cell development^11^,^12^ and germ line protection^13^. Mutations in the p63 gene in humans lead to diseases such as Hay-Wells syndrome^14^, cleft-lip, split-hand/split-foot^15^, ADULT syndrome^16^ and EEC syndrome^14^,^17^. Knockout of p63 in mice leads to truncated limbs and an underdeveloped skin^8^,^18^, resulting in a drastically reduced lifespan. The p63 protein was also reported to be involved in cancer, apoptosis and chemo sensitivity^19^,^20^,^21^.

All members of the p53 family have the same modular architecture with an N-terminal transactivation domain, a 60% homologous core DNA-binding domain (DBD) that is followed by a tetramerization domain and a regulatory C terminus^22^,^23^. Despite the similarity of their DNA-binding domains, p53 and p63 differ in their DNA binding properties^24^; p53 binds to its target DNA in a highly cooperative manner, whereas p63 lacks cooperativity^24^ and consequently binds to its target DNA sequences considerably more weakly. Several isotypes of p63 and p73 have a conserved C-terminal extension of 100 residues, consisting of a SAM domain^24^ and a regulatory domain^25^, that is not present in human p53 and that might be a protein-protein interaction module with regulatory function^26^,^27^. In addition, N-terminally truncated isoforms of p63 and p73 (ΔN) were identified that are not capable of transcriptional transactivation and that have an anti-apoptotic role as antagonists of their full-length counterparts^4^,^28^.

In contrast to p63-DBD, the thermostability of wild type and mutant p53-DBDs has been studied in detail^29^. Many oncogenic mutations within p53-DBD are known to affect its thermodynamic stability and prevent proper folding and DNA binding^29^. Several studies tried to rescue the mutant protein and stabilize the wild-type p53-DBD conformation^30^,^31^,^32^. These attempts and others using semi-rational design^33^ resulted in protein variants with up to 30 °C increased thermal stabilities. More recently, small molecules have been discovered to stabilize p53-DBD in a wild-type conformation^34^,^35^. With a thermal stability of 65 °C (as compared to 43 °C for p53-DBD wt) p63-DBD might serve as a template for a more structure-based stabilization approach. Recent crystal structures of p63-DBD bound to various DNA response elements^36^,^37^ showed that p63-DBD interacts with DNA in a similar manner than p53-DBD. However, no high-resolution structure has been available for p63-DBD in its apo form as well as no information on the dynamics of this protein domain.

Here we present the solution structure of the 26kD p63 DNA binding domain (p63-DBD, residues Ser114-Thr345) solved by NMR spectroscopy. A structural comparison between p63-DBD and a recent complex structure with DNA revealed pronounced structural changes upon DNA binding. A structural comparison with p53-DBD shows a high degree of similarity between the two homologues. Further, we compared the binding specificities of p63-DBD and p53-DBD to various DNA response elements and anti-apoptotic BclxL. The presence of a double salt bridge in p53-DBD leads to increased binding affinity for all response elements and the introduction of this double salt bridge into p63-DBD restores high-affinity binding to p53 promoter sites, highlighting the general role of a double salt bridge for selective DNA recognition within the p53 family members.

## Results

### Solution NMR structure of p63-DBD

**Figure 1:**
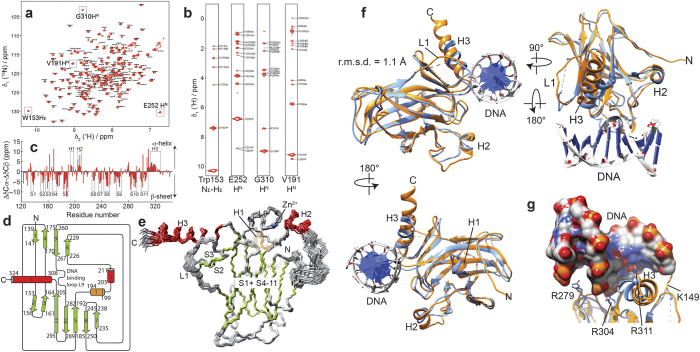
NMR structure of p63 DNA binding domain (DBD). (**a**) 2D-[^15^N, ^1^H]-TROSY spectrum of p63-DBD. (**b**) Example strips taken from a ^15^N-edited 3D NOESY spectrum. (**c**) Secondary chemical shift data. Boxes indicate the location of secondary structure elements in the final structure. (**d**) Topology diagram of p63-DBD based on chemical shift, coupling constants and NOE data. (**e**) Stereo view of the lowest-energy structural ensemble (20 structures) of the p63-DBD with a backbone atom r.m.s.d. of 0.35 Å among ordered residues. The secondary structural elements are labeled. All β-sheets are shown in light green, α-helices in red, the 3_10_ helix in orange and unstructured regions in grey. (**f**) Structural comparison between apo-p63-DBD (determined here, orange) and p63-DBD bound to DNA (blue, pdb id: 3qym)^36^. The two structures show a backbone r.m.s.d. of 1.1 Å. (**g**) Helix 3 undergoes a pronounced change in location upon binding to DNA. In the complex structure, this helix moves towards the DNA by around 3.3 Å. This conformational change leads to a repositioning of the side-chains of Arg 311, which enables specific DNA binding. Other DNA-binding residues, like Arg 279 and Arg304 are being slightly reoriented in the complex with DNA to engage in specific interactions. In the NMR structure Lys 149 is oriented toward the solvent in an extended manner and is thus available for establishing specific contacts to DNA. This crucial side-chain is not resolved in the crystal structure.

In contrast to p53-DBD, p63-DBD can be produced in *E. coli* at 37 °C as a soluble protein in large amounts (see methods section). We were able to obtain up to 20 mg of purified isotope-labeled p63-DBD per liter of M9 medium for our structural studies. Using NMR methods we could further complement our resonance assignment (Fig. 1a)^38^ for all backbone and side chain atoms, with the exception of the segment S233–V238 (S262–V267 according to SwissProt accession code Q9H3D4), where the corresponding backbone amide signals could not be observed in the 2D-[^15^N, ^1^H] TROSY spectrum, most likely due to unfavorable exchange processes. NOE-derived distance restraints extracted from a set of 2D and 3D NOESY spectra (Fig. 1b), chemical shift-derived backbone torsion angle restraints (Fig. 1c) and ^3^J_HNH◽_ and ^3^J_NH◽_ coupling constants were used for structure calculations (Table 1). The final ensemble consisting of the 20 lowest-energy structures (Fig. 1e) is well defined with an RMSD of 0.35 Å for all backbone atoms and 0.79 Å for all non-hydrogen atoms (Table 1). The overall structure of p63-DBD adopts an immunoglobulin fold; i.e. an 11-stranded β-sandwich (Fig. 1de). The sandwich consists of four β-strands (1, 4, 6 and 9) that are exposed to the solvent at one side, and of β-strands 2 and 3 (capped by helix 2) and 5, 7, 8, 10 and 11 on the other, of which at least strands 10 and 11 are shielded from the solvent by the N-terminus. The long loop linking β5 to β6 contains two helices, a 3_10_-helix (helix 1, residues 194–199) and an α-helix (helix 2, residues 205–211). Helix 3 (residues 308–324) is located above β-strands 2 and 3 and represents the main DNA binding interface in the p53-DBD homologue. On the other side of the DNA contact area there is an extended loop region (L9) of 15 residues length; a well-defined and highly conserved structural feature between β-sheets 9 and 10^39^,^40^

### p63-DBD undergoes pronounced conformational changes upon binding to DNA

Our NMR structure of apo-p63-DBD and a previous crystal structure of the p63-DBD-DNA complex^36^ provide the opportunity to analyze structural changes occurring upon binding to DNA (Fig. 1f). The overall immunoglobulin fold is preserved in both structures with a pairwise RMSD of 1.1 Å. In the NMR structure, we can clearly see a 3_10_ helix (H1) consisting of residues 194 to 199 that is not well defined in the crystal structure. Furthermore, loop 1 (L1) is not completely resolved in the crystal structure. In the NMR structure this loop adopts a similar conformation as seen in p53-DBD, where a lysine residue (Lys149) points toward the solvent and is available for interaction with DNA (Fig. 1g). In addition, helix 3 (H3) is one turn longer in solution than in the crystal structure. Dynamics in this C-terminal region of the protein is enhanced (Supplementary Fig. 2), presumably leading to a reduced resolution in the crystal structure. Importantly, there are marked structural differences within the DNA binding interface of p63-DBD. Helix 3, one of the main interaction sites with DNA, experiences changes in its position upon DNA binding of around 3.5 Å (Fig. 1f,g). By this conformational change, helix 3 inserts itself into the major groove of the bound DNA. This results in repositioning of Arg311 side chain for subsequent interaction with the DNA phosphate backbone. Other DNA-binding residues in p63, like R279 and R303, also experience a slight change in position and orientation leading to a specific interaction with DNA (Fig. 1g).

### Optimized packing in the hydrophobic core of p63-DBD leads to high thermal stability

**Figure 2:**
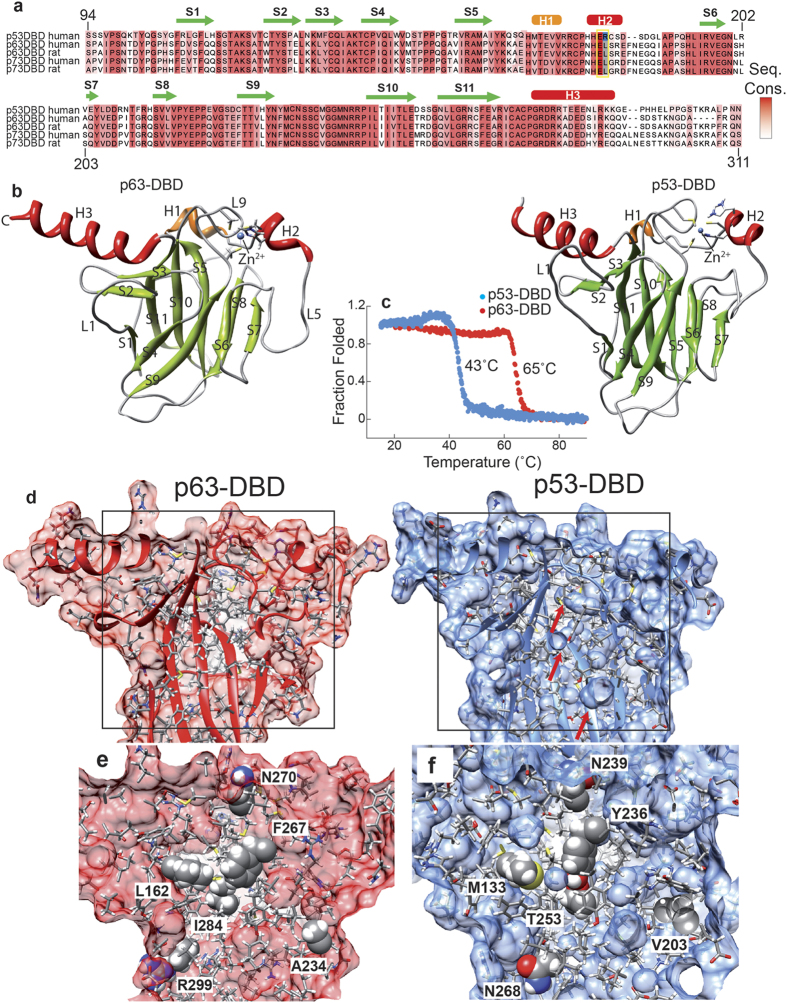
Structural homology and stability of p63-DBD and p53-DBD. (**a**) Multiple sequence alignment of p53-family proteins. Secondary structural elements in p63-DBD are indicated. p53-DBD and p63-DBD share a 55% sequence identity. (**b**) The solution structures of p63-DBD (left) and p53-DBD (right) show the same structural topology and secondary structure content. (**c**) CD-detected thermal unfolding traces of both proteins reveal a large difference in thermal stability between p63-DBD (red, 65 °C) and p53-DBD (blue, 43 °C). (**d**) Packing of amino acid side chains in the hydrophobic core of p63 (red) and p53 (blue). Spheres within p53-DBD indicate unfavorable cavities. (**e**) The protein core of p63-DBD is tightly packed by hydrophobic side chains. Labeled amino acids correspond to positions in p53-DBD that have been mutated by rational design. (**f**) p53-DBD is less tightly packed with hydrophobic amino acids. The indicated amino acids have been mutated to the corresponding residues in p63-DBD in order to enhance protein stability.

**Figure 3:**
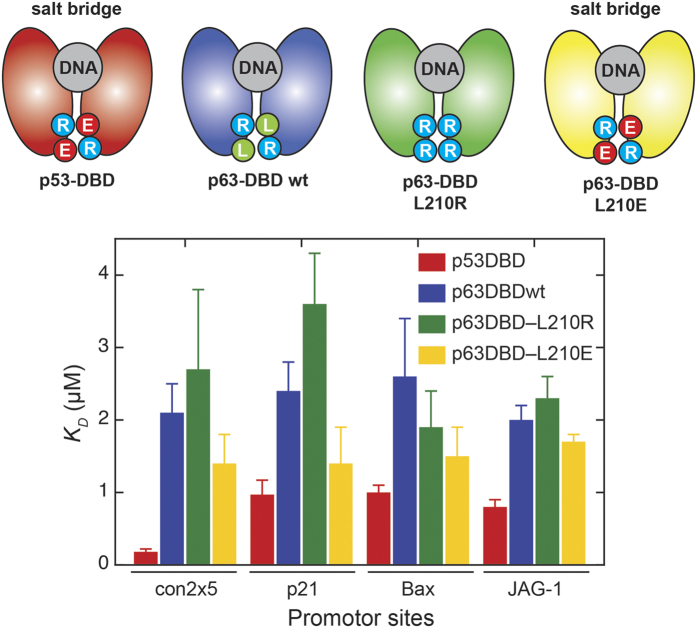
Influence of a double salt bridge on the interaction between p63-DBD and various promotor sites. p53-DBD forms an Arg-Glu’– Arg’-Glu double salt bridge between the two monomers in the dimer. In p63-DBD the glutamate is replaced by a leucine residue, thus reducing its affinity to all DNA promotor sites. The affinity is further reduced by mutation of the leucine to another arginine, but significantly enhanced if mutated to glutamate. Four different promotor sites were used: p53 specific con2 × 5, *p21, Bax* and p63 specific *Jag-1*.

The p53 family of transcription factors shows high sequence conservation between both isoforms and among different species (Fig. 2a). Accordingly, there is high structural conservation among the family members. A particular region of interest is helix 2 (H2) that harbors a Glu-Arg sequence element. These two charged residues form a double salt bridge upon p53-DBD dimerization induced by DNA binding^41^,^42^. In addition to several p53-DBD structures and two p63-DBD structures in complex with DNA^36^,^37^ solved up to date, we here provide additional structural information on apo-p63-DBD in solution. A comparison of these structures shows that both proteins adopt a similar β-barrel immunoglobulin fold with exactly the same number of β-strands and α-helices (Fig. 2b) and a pairwise RMSD of 1.5 Å. The protein cores of both p53 and p63 are compactly folded as proven by {^1^H}-^15^N heteronuclear NOE experiments (Supplementary Fig. 2), which report dynamics in the ns-ps time scale. It adopts low to negative values for very flexible parts of the protein and higher values for rigid and compactly folded parts. In both proteins the folded part is equally rigid with only the termini being more flexible. Despite these similarities in secondary and tertiary structure both proteins differ markedly in their thermal stability. The melting point of p53-DBD with 43 °C is more than 20 °C below that of p63-DBD (65 °C) as monitored by far-UV CD-detected thermal transitions (Fig. 2c). The availability of structural information of both p53-DBD family members allows us to analyze the molecular details of protein stability in each case. Previous work based on mutational data and sequence analysis of more stable p53-DBD orthologs resulted in engineered p53-DBD variants with higher thermodynamic stability^29^,^33^,^40^,^43^,^44^. According to reports by Fersht and colleagues^43^,^44^, most stabilizing mutations within p53-DBD are located in the hydrophobic core. We therefore investigated the packing quality of hydrophobic residues in the protein core of both proteins (Fig. 2d). In order to relax the protein structure and alleviate unfavorable side-chain conformations biased by differences in structure calculation methods, we performed an energy minimization and a 100ps-molecular dynamics simulation run with the p53-DBD NMR structure^40^ and the p63-DBD solution structure presented here. An analysis of both structures indicated that p53-DBD contains cavities in the hydrophobic core (red arrows in Fig. 3d, right panel), whereas these are absent in p63-DBD. Furthermore, in contrast to p63, p53-DBD contains a number of unfavorable polar residues in its core that cannot form pairwise polar interactions. These imperfections in the hydrophobic core of p53 have been discovered and exploited for semi-rational design^33^,^44^ in order to develop p53-DBD variants with enhanced stability for structural studies. Strikingly, most amino acid changes in these stabilized p53 variants represent the same amino acid types as present at the corresponding positions in p63 (Fig. 2e,f). Residues in p53 located at the protein surface got replaced by charged residues (N268D, R299 in p63) and residues located in the hydrophobic core are replaced by more bulky hydrophobic or size-optimized side chains (M133L, L162 in p63; V203A, A234 in p63; Y236F, F267 in p63; T253I, I284 in p63). This result emphasizes the crucial role of a compactly packed hydrophobic core for protein stability.

### The presence of a double salt bridge governs DNA binding specificity

In its active form, p63 binds specifically to DNA as a homo-tetramer, in which each dimeric protein subunit binds to two basically identical DNA half-sites with a linker of varying length^45^. The DNA binding domain of p53 was shown to bind specifically to one half-site of this DNA sequence as a homo-dimer^24^. In previous studies, the lack of cooperativity of DNA binding in p63 was associated with the absence of an inter-dimer double salt bridge (Arg209 and Leu210 instead of Arg180 and Glu181 present in p53)^41^. Further, it was found that this cooperativity affects the binding properties to various promoter sites, leading to either apoptosis or cell-cycle arrest^46^,^47^. We investigated the binding affinities of p53-DBD, p63-DBD and p63-DBD variants where the missing double salt bridge was either restored (L210E) or completely disrupted (L210R). As substrates we used fluorescein labeled (FL) p53 consensus sequence (con2 × 5), the *p21* and *Bax* DNA element and a more p63 specific *Jag-1* response element, whose gene encodes for Jagged1, a ligand of the Notch receptor^48^, linked to cell proliferation and differentiation. p53-DBD exhibits the highest affinity for all tested DNA response elements (Fig. 3), highlighting the role of the double salt bridge for high-affinity binding. p63-DBD shows an almost 10-fold lower affinity for the con5 × 2 p53 consensus sequence and 2-fold lower affinity for the other promoter sequences tested. However, when the double salt bridge was introduced into p63 (L210E), a gain in affinity was observed for all dsDNA sites. The opposite tendency holds true for the L210R variant of p63-DBD (Fig. 3), which most likely disrupts homo-dimerization due to charge repulsion. Among the tested DNA sites, the p63-specific response element of *Jag-1* and *Bax* were less affected by these amino acid substitutions, leading to the assumption that these response elements do not rely on the formation of a double salt bridge.

**Figure 4:**
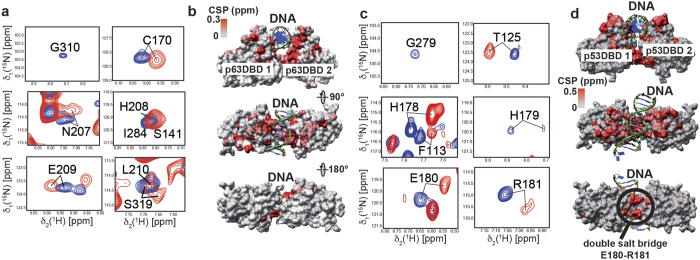
DNA binding of p63-DBD and p53-DBD. (**a**) Titration of ^2^H,^15^N-labeled p63-DBD (blue) with 0.5 eq ds-con2 × 5 DNA (red) monitored with 2D-[^15^N, ^1^H]-TROSY experiments. (**b**) Chemical shift perturbations (CSPs) mapped onto a dimeric structure of p63-DBD bound to a DNA half site (pdb id: 3qym^36^). Regions in p63-DBD that are most affected are colored in red. (**c**) Sample spectra of ^2^H,^15^N-labeled p53-DBD upon titration with 0.5 eq ds-con2 × 5 DNA (blue vs. red contour lines). (**d**) CSPs mapped onto a crystal structure of dimeric p53-DBD bound to a decamer DNA site (pdb id: 2geq)^42^. In addition to the DNA-binding sites in p53-DBD, helix 1 shows strong CSP effects upon DNA binding caused by the formation of a double salt bridge with the second p53-DBD monomer.

To evaluate the DNA-binding properties of both p53-DBD and p63-DBD in solution, we used NMR titration experiments to extract chemical shift differences within both proteins upon the addition of DNA (Fig. 4). We added the double-stranded 16 base-pair con2 × 5 DNA consensus sequence (5′-TTAGGA CATG TCCATC-3′)^45^ stepwise to *U*-[^2^H,^15^N]-labeled p63-DBD or p53-DBD. The 2D-[^1^H;^15^N]-TROSY spectra showed line broadening even at low DNA concentrations (0.1–0.5eq). Only one set of signals with broad lines was observed in the spectra of p53-DBD and p63-DBD (Fig. 4a), strongly suggesting that line broadening is caused by chemical exchange processes in the ms-μs time-scale. Nevertheless, reliable data could be extracted for most amino acids (Supplementary Fig. 1a). These data show that the DNA-binding interface of p63 is very similar to that of p53^42^. It consists of the C-terminal helix 3 as well as the β-turn region (L9) following the β-strand 9. However, we see marked differences between p53-DBD and p63-DBD at the dimerization site, where a double salt bridge in p53 (Glu_180_ Arg_181_/Glu_180_’Arg_181_’) mediates protein-protein contacts. Large chemical shift perturbations in this region could only be observed with p53-DBD and are absent or less pronounced in p63-DBD (Fig. 4b). The NMR data are in good agreement with the affinities of p53-DBD and p63-DBD to DNA promoter sites where the double salt bridge generally leads to an increase in affinity.

### The binding sites of p63 and p53 to BclxL are homologous

**Figure 5:**
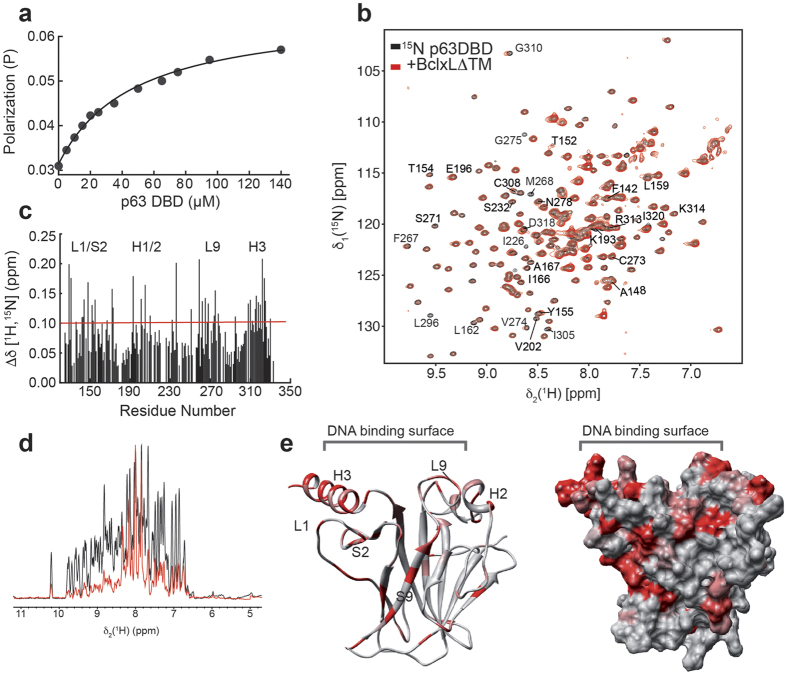
Interaction of p63-DBD with BclxL. (**a**) Fluorescence polarization of fluorescein-labeled BclxLΔTM (without its insoluble transmembrane helix) upon titration of p63DBD yielding a *K*_*D*_ of 38 ± 18 μM. Triplicate measurements were done to estimate standard error. (**b**) 2D-[^15^N; ^1^H] TROSY experiments of 200 μM p63-DBD alone (black) and in presence of a twofold excess of BclxLΔTM (red). (**c**) ^1^H, ^15^N averaged chemical shift perturbations (CSPs) of p63-DBD extracted from the spectra shown in (**b**). (**d**) Projection along the ^1^H dimension taken from 2D-TROSY spectra shown in (**b**) exhibit a pronounced decrease in signal intensity upon complex formation with BclxL (black: p63-DBD; red: in complex with BclxL). (**e**) CSPs mapped onto the solution structure of p63-DBD ranging from light grey (no effect) to red (>mean CSP + standard error).

As a response to apoptotic stimuli, p53 translocates to the outer mitochondrial membrane where it activates a transcription-independent apoptosis pathway^49^. The mechanism of this pathway is not fully understood, but it is known that p53 is capable of binding to several members of the Bcl2 family, such as BclxL^50^. This binding is suggested to activate the pro-apoptotic members Bax,^51^ Bak^52^ and Bad^53^ by promoting dissociation of anti-apoptotic members like Bcl2 and BclxL^54^. Recent studies revealed the binding site of BclxL to p53-DBD^50^,^55^,^56^, showing it to be different from the binding site for pro-apoptotic Bak^55^. Considering the high structural and functional similarity of p53- and p63-DBDs, we decided to characterize the binding of p63-DBD to BclxL. We used chemical shift perturbation experiments to probe the interaction between ^15^N-labeled p63-DBD and BclxL (Supplementary Fig. 1b). Our titration experiments (Fig. 5) show that the DNA binding site in p63-DBD is mediating interaction with BclxL, similar to what has been observed previously for p53^50^,^57^. The induced chemical shift perturbations within p63-DBD are clustered around its DNA binding site. Additionally, some signals originating from residues at the DNA binding site completely disappear upon complex formation. This behavior is indicative of the formation of a larger complex and binding kinetics on the ms-μs time scale, which is often observed for interactions in the μM-affinity range. In line with the NMR data, a binding affinity between BclxL and p63-DBD of 38 ± 18 μM was obtained by fluorescence polarization (FP) experiments, which is lower than for p53-DBD (5.5 μM)^50^. These differences in binding affinity might also reflect a lower pro-apoptotic potential of p63 as compared to p53.

## Discussion

We have determined the solution structure of apo p63-DBD using NMR spectroscopy (Fig. 1) and characterized the interaction with various DNA binding sites (Figs 3 and 4) and the anti-apoptotic Bcl2-familiy member BclxL (Fig. 5). p63-DBD adopts an almost identical structure as p53-DBD (Fig. 2b). However, there are differences in DNA binding specificity and in its thermodynamic stability that render p63 an attractive target to understand the structural basis of DNA binding specificity and protein stability among the p53 protein family.

Despite the availability of structural information and DNA binding data, the target gene specificity of p63 is not yet clear. Transactivation of p53 target genes regulating cell-cycle arrest by p63 have been demonstrated by *in vivo* DNA binding assays^58^ and ChIP experiments with E1A-expressing mouse embryo fibroblasts revealed p63 binding to pro-apoptotic p53 target genes as well as *MDM2*, whose expression leads to p53 degradation^59^. Another factor is the occurrence of truncated p63 variants lacking their N-terminal transactivation (TA) domain (ΔNp63). These variants adopt a transcription-repressive role^4^ and are able to inhibit transcription of p53 target genes. Furthermore, p63 and p73 contain a C-terminal SAM^26^ and regulatory domain^25^ that influence their capability to transcribe target genes. Another layer of complexity is added by the presence^13^ of different oligomeric states, where the dimer seems to be the transcription-inactive and the tetramer the active species. The formation of tetramers and the resulting p63 DNA-binding activation is promoted by phosphorylation^13^.

It has been demonstrated that p63 and p73 knockout does not affect transcription of genes regulating cell-cycle arrest, like *p21*^59^. However, p63 and p73 seem to be required for efficient induction of pro-apoptotic p53-dependent genes. These results indicate that apoptosis-related genes are specifically regulated within the entire p53 family. On the other hand, p63-mediated epithelial development is not affected by p53 knockout. Our DNA binding assays (Fig. 3) indicate interaction of p53 and p63 with DNA response elements involved in cell cycle arrest, apoptosis and development to varying extents. It has been previously reported that p53 DNA binding relies on the establishment of a double salt bridge^41^, a specific feature of p53. We therefore introduced a double salt bridge into p63-DBD and assayed the change in binding affinity compared to wild-type p63. The assumption is that p63-DBD capable of forming a salt bridge binds to p53-dependent genes with higher affinity, whereas less p53-dependent genes would show weaker or no dependence on the presence of the double salt bridge. In line with published *in vivo* data^59^ the largest change in affinity could be observed for *p21* RE, confirming a less dominant role of p63 in the induction of cell cycle arrest. A p53-specific consensus site (con2 × 5) showed a similar dependence on the presence of the double salt bridge. The involvement of the double salt bridge in cooperative high-affinity DNA binding could also be confirmed by NMR chemical shift perturbation studies with p53-DBD *versus* wt-p63-DBD (Fig. 4). These data demonstrate that p53 has evolved for high-affinity binding to DNA and thus needs to be tightly regulated by a carefully balanced protein production and degradation equilibrium. In contrast, binding to the *Bax* RE is not significantly affected by the double salt bridge, confirming that pro-apoptotic genes might be less p53-specific. The p63-dependent RE of *Jag-1*^48^, a gene involved in cell differentiation and proliferation, shows a similar salt-bridge independent affinity pattern as *Bax*. Recent studies showed that p63 is less prone to degradation via the ubiquitin-proteasome pathway^60^, due to lower affinity between Mdm2 and p63. It is therefore likely that the cellular concentration of p63 is higher than of p53, enabling p63 to efficiently bind to DNA elements and even compete with p53.

The importance of p63 in the induction of apoptosis is underlined by its interaction with the anti-apoptotic protein BclxL (Fig. 5). The interaction surface between both proteins consists of the positively charged DNA-binding interface in p53/p63-DBD (Supplementary Fig. 3) and a negatively charged surface area in BclxL^50^,^57^. As previously shown for p53, direct interaction with Bcl2 proteins induces rapid mitochondrial apoptosis^49^,^50^,^54^,^55^,^56^ in a transcription-independent manner. In order to fulfill its role in limb and epithelial development protection p63 might utilize both direct and indirect apoptosis pathways. The apoptotic potential of p63 is also in line with its role in female germ line protection and its strong expression pattern in oocytes^13^, where it is involved in DNA damage-induced oocyte death, independent of p53.

Compared to p53, p63-DBD shows a more than 20 °C increased thermal stability (Fig. 2c). A variety of cancer-related p53-DBD point mutations lead to a significant destabilization of the already fragile protein, prohibiting proper folding and gene transactivation. The basis for the low stability of p53-DBD is unfavorable packing of its hydrophobic core, caused by isolated polar side chains and internal cavities (Fig. 2f). There have been tremendous efforts to design p53-DBD variants with increased stability for structural studies^31^,^32^,^33^,^43^,^44^ that might eventually be used for gene therapeutic approaches^61^. For one of these studies, our p63-DBD solution structure served as a template for the semi-rational design of p53 variants^44^. Furthermore, small-molecules that stabilize the active conformation have been reported for p53 34,35. Hydrophobic packing quality is significantly improved in p63-DBD (Fig. 2e) and optimized p53 variants consequently show the same amino acid type at critical positions as found in p63 or more stable p53 orthologues. Due to its high thermodynamic stability, p63 DNA binding activity is less likely to be diminished by single point mutations in its protein core, as shown for the p53 case. Furthermore, no destabilizing mutations within p63-DBD have been found in cancer tissues^19^. It has therefore been suggested that p63 acts as an oncogene and not as a tumor suppressor. The oncogenic form of p63 is the ΔN-variant lacking its N-terminal transactivation domain. This variant can still bind to DNA elements and compete with p53 and full-length p63 to suppress apoptosis^62^. Mutations in p63 have been identified in developmental diseases, such as EEC^63^ and ADULT^64^, affecting the DNA-binding capability and not protein stability. So far, it is not clear what factors govern the binding specificity and cellular functions of the individual members of the p53 family. Even though plenty of information is available for p53, p63 and in particular p73 are far less well explored. Future structural and functional studies are required to clarify these open questions.

## Methods

### Protein expression and purification

The preparation of protein samples of p63-DBD has been described elsewhere^38^. In brief, p63-DBD (amino acids 153–388) cDNA was sub cloned into a modified pQ40 vector (Qiagen). Proteins were expressed in *Escherichia coli* BL21 (DE3) at 37 °C. A truncated p63-DBD construct consisting of only structured residues lacking flexible tails was chemically synthesized (Gene Art) and cloned into a pET28a vector system (Merck Biosciences). For purification of p63-DBD, the soluble lysate was loaded onto a cation-exchange column and eluted with a linear KCl gradient (0–0.5 M) or onto a Ni-NTA column and eluted with a linear gradient from 20 to 500 mM imidazole. A heparin column (GE Healthcare) was used for further purification. After His-tag removal with Thrombin, final purification was achieved by size-exclusion chromatography with a Superdex75 column (GE healthcare) equilibrated with 50 mM sodium phosphate (pH 7.2), 150 mM NaCl, and 5 mM β-mercaptoethanol. For NMR studies, p63-DBD samples were dialyzed against 50 mM phosphate buffer (pH 6.8), 150 mM KCl and 5 mM DTT and concentrated to 0.7–1.5 mM using Amicon centrifugal devices (Millipore).

A truncated construct of human BclxL lacking the trans membrane domain (residues 1 to 212), so-called BclxLΔTM (Refs 50, 65), was constructed by PCR based deletion in a pET28a expression vector (Novagen). Expression was done in *E.coli* BL21(DE3) and purification was achieved by Nickel-NTA, Anion-exchange and size exclusion chromatography^50^.

Buffer exchange to 50 mM potassium phosphate pH 6.8, 150 mM KCl, 5 mM DTT was achieved by passage over a G-60 column (GE healthcare). Analysis of all samples using SDS-PAGE indicated high purity (>95%) and confirmed the correct molecular mass.

### Circular dichroism (CD) spectroscopy

CD spectroscopic experiments were done with a Jasco J-715 spectropolarimeter (Jasco, Gross-Umstadt, Germany). For CD spectra and thermal transition experiments, protein concentrations of 10 μM in 10 mM sodium phosphate pH 7.2, 1 mM TCEP were used. Thermal transition experiments were recorded from 20 to 90 °C with a heating rate of 60 K/h. Data were fitted with a two-state folding model as described previously^66^. A response of 2 s, a bandwidth of 5 nm and a cuvette of 1 mm path length was used for all measurements.

### Fluorescence polarization experiments

Fluorescence polarization experiments were done with a BMG Polarstar Galaxy (BMG Labtec, Offenburg, Germany) multimode plate reader. 100 nM of ds 5′-fluorescein-labeled DNA response elements or N-terminally labeled BclxLΔTM (without trans membrane helix) were titrated with increasing amounts of p63-DBD/p53-DBD in 10 mM sodium phosphate pH 7.2, 1 mM DTT. Data were fitted with a one-site or cooperative binding model using ProFit (Quantum Soft, Uetikon am See, Switzerland) as described previously^41^.

### NMR experiments

All spectra were recorded at 303 K on Bruker DMX600, DMX750 and Avance900 spectrometers. Backbone sequential assignment was completed using standard triple resonance experiments based on earlier results^38^.Aliphatic side chain assignments were done using H(C)CH- and (H)CCH-COSY^67^ experiments. The use of ^13^C-TOCSY-sidechain experiments was beneficial only for the most slowly relaxing amino acids. Stereospecific assignments and the resulting χ_1_ rotamer assignments were determined for 106 of the 156 prochiral C^β^Η_2_ protons and for the C^γ^Η_3_ groups of all 17 valine residues. Assignments of χ_1_ rotamers were also available for 13 of 14 isoleucine residues and 12 of 18 threonine residues. Assignments of χ_2_ rotamers were made for 12 of 14 isoleucine and 8 of 10 leucine residues. Distance restraints were derived from a set of five 3D-NOESY spectra, including the heteronuclear edited NNH- and CNH-NOESY spectra^68^ in addition to conventional ^15^N- and ^13^C-HSQC-NOESY spectra. NOE spectra were acquired using the following parameters (proton frequency, resolution in F1, F2, F3, mixing time, total experiment time): HNH-NOESY (900 MHz, 15 N 39 Hz, 1 H 31 Hz, 1 H 9 Hz, 70 ms, 13 h), HNH-NOESY (600 MHz, ^1^N 37 Hz, ^1^H 56 Hz, ^1^H 7 Hz, 100 ms, 10 h), CNH-NOESY (900 MHz, ^15^N 39 Hz, ^13^C 31 Hz, ^1^H 9 Hz, 80 ms, 168 h), NNH-NOESY (750 MHz, ^15^N 27 Hz, ^15^N 20 Hz, ^1^H 10 Hz, 80 ms, 123 h), HCH-NOESY (900 MHz, ^13^C 170 Hz, ^1^H 74 Hz, ^1^H 9 Hz, 70 ms, 67 h). In addition, backbone dihedral angle restraints (as predicted by the program TALOS^69^ and verified with NOE data), sidechain dihedral restraints (derived from NOE data and qualitative evaluation of ^3^J(N-H^β^) couplings), ^3^J (H^N^-Hα-coupling constant restraints and hydrogen bond restraints (implemented as pseudo-covalent bonds^70^ were used as input for structure calculation. Stereospecific assignment and NOE restraint refinement was carried out by comparison of experimental and back calculated ^15^N-NOESY-HSQC, NNH- and CNH-NOESY spectra (in house software). This procedure facilitated the adjustment of most side chain rotamers. Structures were calculated with XPLOR-NIH using standard protocols with minor modifications as described elsewhere^71^. For the final set, 200 structures were calculated and 20 chosen on the basis of lowest overall energies. An average structure was calculated and regularized with experimental restraints yielding a representative of the structural ensemble (model 1 in the protein data base entry  rmn, used here for all NMR figures). Titration experiments were monitored with ^1^H-^15^N TROSY experiments. 400 μM of triple (*U*-^2^H, ^13^C, ^15^N) or double (*U*-^13^C, ^15^N) labeled p63DBD was titrated with an at least 2-fold excess of unlabeled BclxLΔTM (without the trans-membrane helix) in 1 mM sodium phosphate pH 7.2, 5 mM DTT or a 1/5 molar ratio of con2 × 5 double-stranded consensus DNA in 1 mM sodium phosphate pH 7.2, 100 mM NaCl, 5 mM DTT. Titration experiments with 100 μM U-^2^H,^15^N p53-DBD were done in the same buffer and upon addition of 50 μM ds-con2 × 5. The heteronuclear NOE was derived using standard experiments and a presaturation time of 2 seconds for 750 MHz and 3 seconds for 900 MHz.

## Additional Information

**Accession codes**. The coordinates of the p63 DBD structural ensemble have been deposited at the Protein Data Bank under accession code 2 rmn and the chemical shift assignments as well as a restraint table can be downloaded from the BMRB data bank (accession code 11012).

**How to cite this article**: Enthart, A. *et al*. Solution structure and binding specificity of the p63 DNA binding domain. *Sci. Rep.* **6**, 26707; doi: 10.1038/srep26707 (2016).

## Supplementary Material

Supplementary Information

## Figures and Tables

**Table 1:** NMR structural statistics of apo p63-DBD.

	SA		<SA>_r_	
RMSD from distance restraints (Å) ^2^				
all (554)	0.029 ± 0.003		0.027	
intra-residue (10)	0.023 ± 0.008		0.025	
inter-residue sequential (42)	0.021 ± 0.002		0.023	
medium range (89)	0.033 ± 0.001		0.033	
long range (335)	0.032 ± 0.001		0.029	
hbond (78)	0.012 ± 0.001		0.010	
RMSD from dihedral restraints (652)	0.12 ± 0.01		0.11	
RMSD J-coupling restraints (Hz) (48)	0.82 ± 0.02		0.78	
H-bond restraints average (Å/deg) (78)	2.12 ± 0.2/16.0 ± 7.2		2.13 ± 0.1/13.3 ± 6.6	
H-bond restraints min-max (Å/deg)	1.42–2.58/5.3–38.2		1.83–2.56/1.5–35.7	
Deviations from ideal covalent geometry				
Bonds (Å × 10^−^3)	4.88 ± 0.18		7.34	
Angles (deg)	0.73 ± 0.03		0.73	
Impropers (deg)	3.58 ± 1.08		1.53	
Ramachandran Map regions (%)^3^	88.9/9.6/0.7/0.8		88.4/10.6/0.0/1.0	
Atomic RMSD (Å)^4^ in Structured areas^6^	**SA vs. <SA>**		**SA vs. <SA>_r_**	
	**Backbone**	**All**	**Backbone**	**All**
	0.35 ± 0.06	0.79 ± 0.09	0.42 ± 0.09	0.90 ± 0.18

^1^SA, 20 final structures; <SA>, mean structure; <SA>_r_, the structure obtained by regularising the mean structure under experimental restraints.

^2^Numbers in brackets indicate the number of restraints of each type.

^3^Determined using the program PROCHECK 3.5.1.

^4^Based on heavy atoms superimpositions.

^5^Structured areas: 164–251, 263–292 and 300–362.

^6^RMSD for superimposition over ordered residues.
