# Supplementary material for: Solution structure and binding specificity of the p63 DNA binding domain
Source: Sci Rep. 2016 May 26;6:26707. doi: 10.1038/srep26707 (PMC4880913; doi:10.1038/srep26707)
Supplement: Supplementary Information [file srep26707-s1.doc]

**Supplementary information to:**

**Solution structure and binding specificity of the p63 DNA binding domain**

Andreas Enthart^a,b,f^, Christian Klein^d^, Alexander Dehner^a,2^, Murray Coles^e^, Gerd Gemmecker^a^, Horst Kessler^a,b^, and Franz Hagn^a,b,c,1^

^a^Center for Integrated Protein Science Munich (CIPS^M^) at the Department Chemie, Technische Universität München, 85747 Garching, Germany

^b^Institute for Advanced Study, Technische Universität München, 85748 Garching, Germany

^c^Helmholtz Zentrum München, Institute of Structural Biology, 85764 Neuherberg, Germany

^d^ Roche Pharmaceutical Research & Early Development, Roche Innovation Center Zurich, CH-8952 Schlieren, Switzerland

^e^Department of Protein Evolution, Max-Planck-Institute for Developmental Biology, Tübingen, Germany

^f^present address: TBK Patentanwälte, Munich, Germany

^1^ To whom correspondence should be addressed: [franz.hagn@tum.de](mailto:franz.hagn@tum.de)

^2^ Deceased July 23, 2012

**Supplementary Fig. 1**


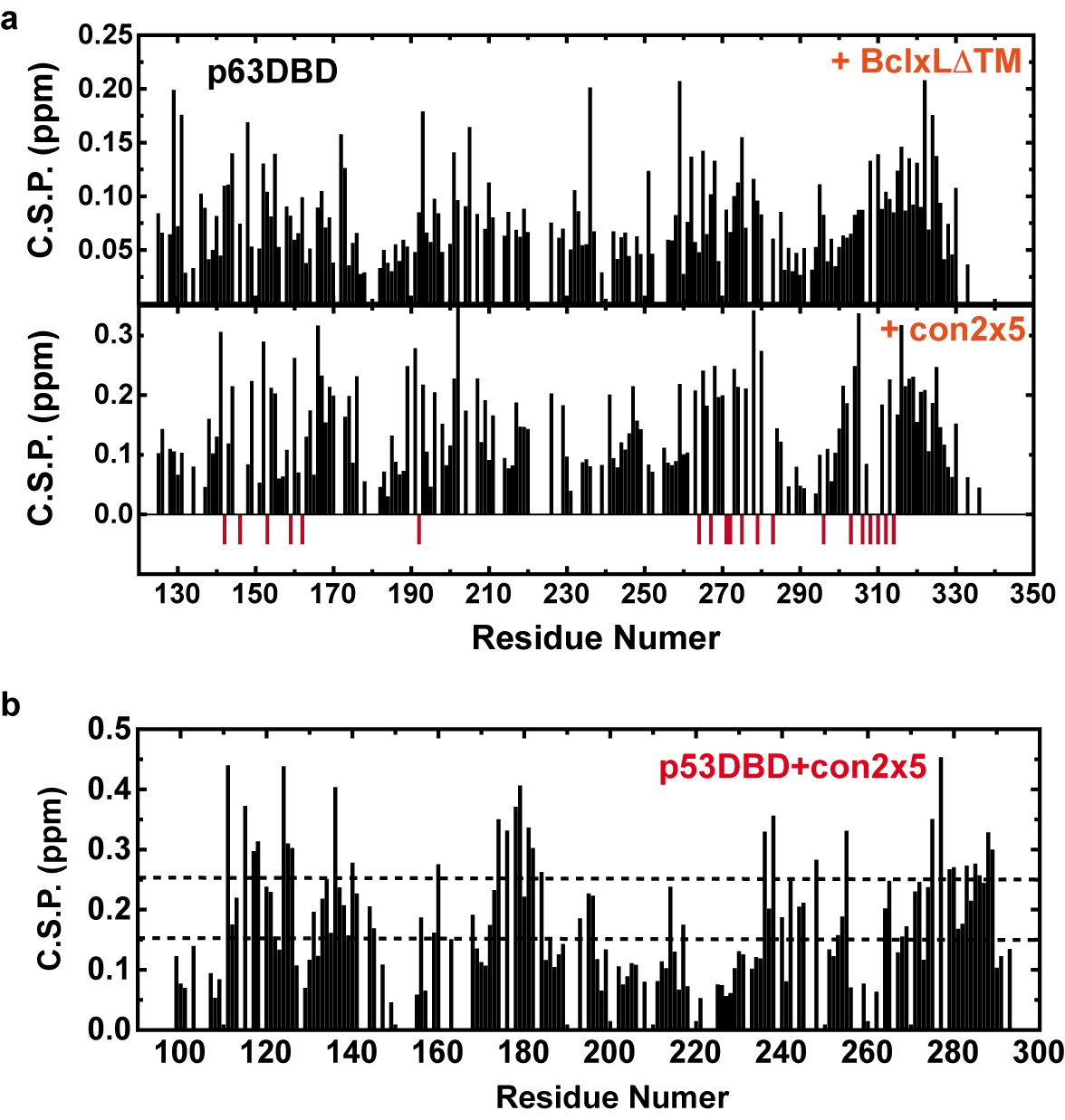


**Supplementary Fig. 1:** Chemical shift perturbations (C.S.P.) in p53-DBD and p63-DBD upon the addition of con2x5 DNA sites and BclxL.

**Supplementary Fig. 2**

**
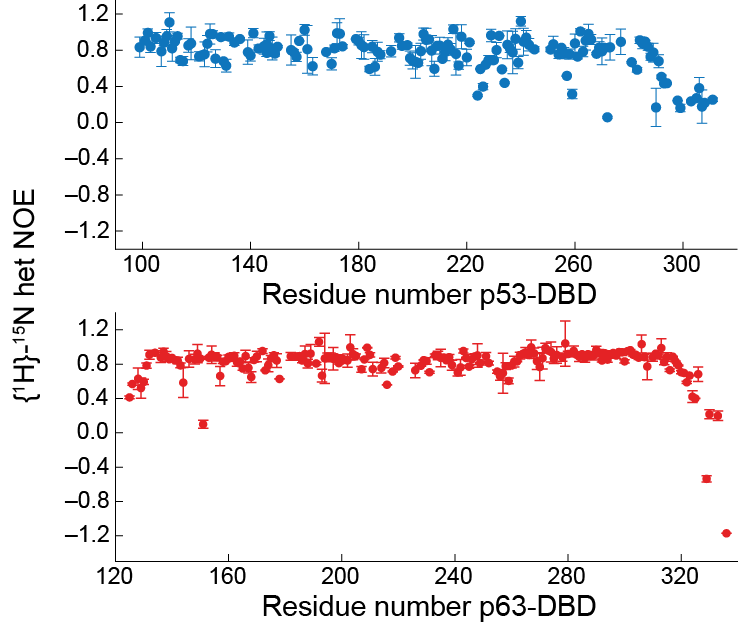
**

**Supplementary Fig. 2:** {^1^H}-^15^N-heteronuclear NOE of p53 (blue) and p63-DBDs (red). Values around 0.8 indicate rigid secondary structure, lower values are indicative of enhanced flexibility. Both proteins are rigid throughout their sequence with only residues located at the termini being more mobile (low or negative hetNOE).

**Supplementary Fig. 3**

**
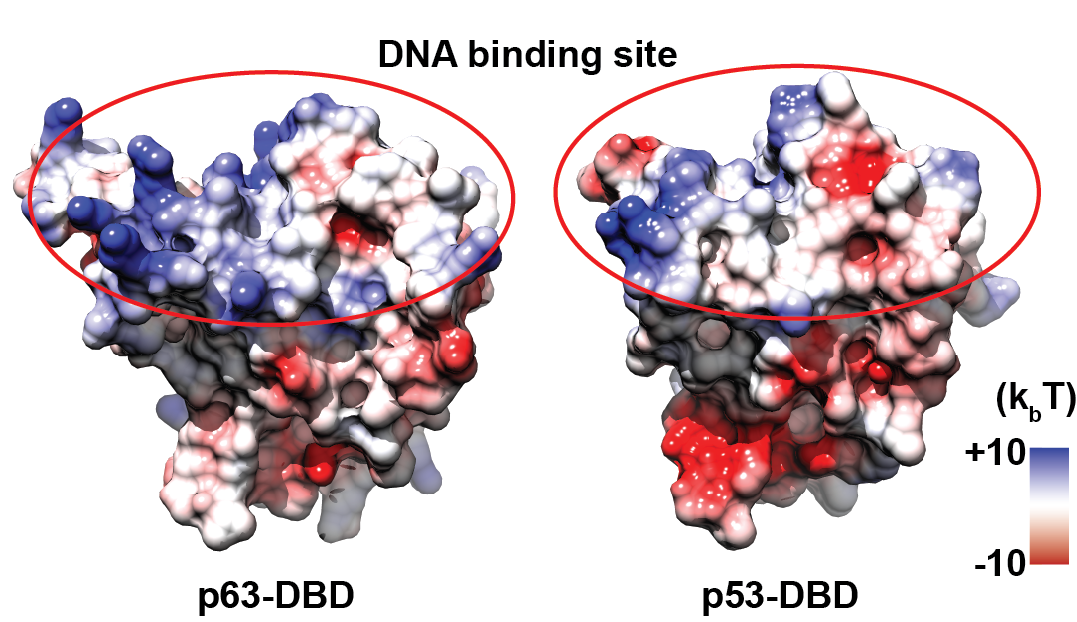
**

**Supplementary Fig. 3:** Surface charge of p63-DBD and p53-DBD. Both proteins exhibit a similar charge distribution.
